# Paralytic ileus in 57 cows – symptoms, diagnosis and treatment

**DOI:** 10.1186/s13028-025-00817-6

**Published:** 2025-06-11

**Authors:** Ueli Braun, Christian Gerspach, Rahel Scheiwiller, Monika Hilbe, Karl Nuss

**Affiliations:** 1 Department of Farm Animals, Vetsuisse Faculty, University of Zurich, Zurich, Switzerland; 2 Institute of Veterinary Pathology, Vetsuisse Faculty, University of Zurich, Zurich, Switzerland

**Keywords:** Cattle, Intestines, Paralytic ileus

## Abstract

**Background:**

Paralytic ileus (PI) also known as functional ileus or adynamic ileus occurs when intestinal motor activity is impaired. Cessation of ingesta passage leads to the accumulation of fluid and gas causing intestinal dilatation. With this type of ileus, intestinal transit is functionally impaired in the absence of a physical obstruction. The present retrospective study describes the diagnosis, treatment and outcome of 57 cows with PI.

**Results:**

Colic occurred in 43.9% of the cows, and intestinal and rumen motility was reduced or absent in 92.9% (52/56) and 82.5% (47/57) of the cows, respectively. Ballottement and/or percussion and simultaneous auscultation on the right were also positive in 82.5% of the cows. Faecal output was minimal or absent in 94.7% (54/57) of the cows, and dilated small intestines and occasionally large intestines were palpated transrectally in 57.1% (32/56). The principal laboratory abnormalities were hypokalaemia (89.4%, 51/57), hypocalcaemia (87.5%, 35/40), hypermagnesaemia (77.5%, 31/40), positive base excess (57.4%, 27/47), acidosis (55.3%, 26/47) and hypercapnia (53.2%, 25/47). Ultrasonography in 50 cows revealed dilated small intestines with reduced or absent motility. Eleven cows had received medical treatment alone, 45 had undergone right flank laparotomy and one was euthanized immediately after clinical examination. Fifty-four (94.7%) cows were discharged and three (5.3%) were euthanized.

**Conclusions:**

Paralytic and mechanical ileus are difficult to differentiate using non-invasive methods, which impacts treatment decisions. The prognosis of cattle with PI is good with adequate treatment.

## Background

Ileus can essentially be divided into two [1] or three types in cattle [2]. German textbooks differentiate between mechanical and paralytic ileus [1], while in American textbooks [2], the first type includes the physical blockage of ingesta passage by obstructions such as bezoars and compression of the intestine by disorders such as fat necrosis. The second type of ileus includes physical obstruction of the intestinal lumen and infarction of the affected bowel section because of volvulus, intussusception, or strangulation of the intestine. Purely functional obstruction of the intestine occurs in the third type of ileus. Functional obstruction is also called paralytic ileus (PI) [3], adynamic ileus [2] and pseudo-obstruction [4] and clinically mimics mechanical obstruction [4] in the absence of a mechanical obstruction or compression [4]. In cattle with PI, impaired intestinal motility and delayed passage of ingesta lead to the accumulation of large volumes of liquid ingesta and gas and enlargement of the affected section or the entire intestinal tract [3]. Local factors associated with the intestinal wall or afferent intestinal nerves, or systemic factors associated with the central nervous system, play a role in the aetiology [3]. Causes include peritonitis, stretching of the intestinal wall after mechanical ileus, excessive manipulation of the intestine during surgery, disturbances in acid-base balance, hypocalcaemia, hypokalaemia and toxaemia [2, 3]. Other causes are stretching or tearing of branches of the vagal nerve in cattle with abomasal torsion, mesenteric diseases, such as abscess, haematoma, fat necrosis and tumours, occlusion of mesenteric blood vessels, and severe infection with *Oesophagostomum radiatum* [3]. Paralytic ileus can also occur after enteritis [2].

The clinical signs of PI and mechanical ileus are similar; the main signs are abnormal general condition, reduced or absent faecal output, abdominal dilatation, positive ballottement and/or percussion and auscultation on the right side and an increase in the number of enlarged small and large intestinal loops [2, 4]. Auscultation of the right flank may reveal fluid tinkling sounds rather than the normal borborygmi. Signs of colic may also be present [4].

A definitive diagnosis of PI usually entails laparotomy to rule out causes of mechanical ileus [3]. In theory, laparotomy is not required for the treatment of cattle with PI because mechanical obstruction is not present. In practice, however, laparotomy is usually needed because PI and mechanical ileus often cannot be differentiated based on clinical, ultrasonographic and laboratory findings. Medical treatment of PI is centred on systemic fluid therapy, intravenous administration of calcium and pain control [4]. Various drugs including neostigmine and metoclopramide have been used to stimulate intestinal motility in horses [2], but the effect of parasympaticomimetic drugs in cows with PI is equivocal [3], and metoclopramide is no longer approved in Switzerland. The use of laxatives has been described [4] but in our experience, laxatives are contraindicated in any type of ileus because they increase intestinal dilatation and thus enhance the reflex inhibition of intestinal motility. Laparotomy is required in cows with persistent colic, abdominal dilatation, a heart rate of > 100/min, little or no faecal output, positive ballottement and/or percussion and simultaneous auscultation on the right side and abnormal abdominal fluid [5] even when a mechanical ileus cannot be ruled out. Surgical decompression of the affected bowel is rarely required in cows with PI [4], but when needed, serves to abolish reflex inhibition of intestinal motility caused by intestinal dilatation. Reflex inhibition is a response to dilatation of bowel segments which causes extreme stretching of the mural tension and pain receptors. Although laparotomy does not serve to relieve a mechanical blockage, defaecation soon after surgery is common [4], presumably because manipulation of the bowel stimulates intestinal motility.

To our knowledge, PI of cattle has only been dealt with in textbooks [2–4, 6]. We were unable to find studies of cows with PI. Therefore, the goal of this study was to describe the clinical findings, treatment and outcome in 57 cows with PI.

## Methods

The medical records of 57 cattle diagnosed with PI between 1990 and 2017 at the Department of Farm Animals, University of Zurich, were analysed. The present work is based on a Master thesis [7].

### Inclusion and exclusion criteria

Only medical records of cattle that were a minimum of one year of age and had PI at the time of admission were included. Twelve cows with PI were excluded because their findings were published previously; they included 5 cows with acute traumatic reticuloperitonitis, 5 cows with toxic mastitis, one cow with a type 3 abomasal ulcer and one with a type 4 abomasal ulcer.

### Clinical examination

All cows underwent a standard clinical examination [8, 9]. Each cow was observed for signs of pain, which were judged as mild, moderate or severe as described [9, 10].

### Laboratory analyses

The collection and examination of blood, urine and rumen fluid were done as described [11].

### Ultrasonographic examination of the abdomen

The abdomen of 50/57 cattle was scanned from the right side as described [12].

### Diagnosis

A tentative diagnosis of ileus based on the clinical examination was made when the main signs were characteristic of ileus, which included signs of colic, reduced or absent faecal output and progressive deterioration in general condition [1]. Paralytic ileus was suspected when signs of ileus were accompanied by a severe illness such as acute mastitis or severe metabolic disease, which were assumed to be the cause of the ileus. The tentative diagnosis of PI was confirmed when the cows responded to medical treatment alone (*n* = 11) or when dilated sections of small and possibly large intestines were seen during laparotomy and/or at postmortem in the absence of a mechanical cause such as intussusception, strangulation, incarceration or volvulus (*n* = 46).

### Medical treatment of 11 cows that were not operated

Medical treatment of the 11 cows that were not operated included systemic fluid therapy, pain control, prokinetic drugs and calcium borogluconate. Calcium, potassium chloride and sodium phosphate were administered via an orogastric tube depending on the serum electrolyte concentrations (details see Results section).

### Laparotomy and postoperative treatment of 45 cows

Right-flank laparotomy was carried out in standing cattle. In one cow that had difficulty standing, the surgery was done in left lateral recumbency. Proximal paravertebral anaesthesia was most commonly used. A 25 to 30 cm incision was made in the mid-paralumbar fossa, and the muscles, fasciae and peritoneum were incised with a scalpel. The abdominal cavity was explored in a standardized fashion [13, 14]. After systematic abdominal exploration, the section of small intestines that was dilated was exteriorized and examined visually and palpated. Finally, the ileocaecal junction, the caecum and the spiral colon were inspected for signs of obstruction. After meticulous exploration and exclusion of any other source of ileus, the intestines were relocated into the abdomen. In a limited number of cases of jejunal and caecal paralysis and dilatation, the bowel content was massaged into the caecum and removed by caecotomy. An antibiotic, most commonly amoxicillin, was infused into the abdomen in 1 L of isotonic saline solution. The peritoneum and the muscle layers were then closed in three layers with synthetic, absorbable, braided suture material (PolysorbTM, USP 2, taper needle, Covidien-Medtronic, Neustadt, Germany) using a simple continuous suture pattern. A modified mattress suture pattern was used to close the subcutaneous tissues (PolysorbTM, USP 0, cutting needle, Covidien-Medtronic) and skin staples were used to close the skin.

The cows that were successfully operated and subsequently discharged were fasted for at least 24 h postoperatively before feeding was gradually resumed. Postoperative treatment was essentially the same as in the non-operated cows with the exception that all operated cows received antibiotics (details see Results section).

### Euthanasia and postmortem examination

Cows that were in a serious clinical condition [9, 10], or when the owner did not consent to surgery, were euthanized using pentobarbital (80 mg/kg body weight, administered intravenously, Esconarkon, Streuli Pharma, Uznach, Switzerland) after the initial examination. Cows were euthanized intraoperatively when lesions associated with a very poor prognosis were seen or complications occurred, and postoperatively when the clinical condition deteriorated. All cattle that were euthanized underwent postmortem examination.

### Statistics

The program SPSS Statistics 26.0 (IBM Corp. 2017, USA) was used for analysis. Frequencies were determined for all variables, and the Shapiro-Wilk test was used to test the numerical data for normality. Means ± standard deviations were calculated for normally distributed data and medians for non-normally distributed data. In addition, the 95% confidence intervals (CI) were calculated for the means and medians. The variables heart rate and rectal temperature over time (days 0 to 7) were analysed using the general linear model choosing ANOVA with repeated measures and replacing polynomial contrasts with difference. A value of *P* < 0.05 was considered significant.

## Results

### Cattle and history

Fifty-three of the 57 cattle (93.0%) were cows and four (7.0%) were heifers. For brevity, all are referred to as cows. They ranged in age from 1.3 to 12.0 years (median, 95% CI, 5.5, 4.0-6.1 years). The breeds included Swiss Braunvieh (34/57, 59.6%), Swiss Fleckvieh (12/57, 21.0%), Holstein (9/57, 15.8%), Angus (1/57, 1.8%) and Braunvieh x Limousin cross (1/57, 1.8%). Three cows were suckler cows and the others were dairy cows. Twenty-two (38.6%) were pregnant, 33.3% (19/57) were open and in 28.1% (16/57), the reproductive status was not recorded. The gestational age of the 22 pregnant cows ranged from 6 to 41 weeks (mean ± sd, 95% CI, 22.3 ± 10.3, 18–27 weeks). The date of the last calving was known for 32 cows and was 1 to 22 weeks before admission to our clinic (median, 95% CI, 9.0, 3–13 weeks). The duration of illness before admission ranged from 2 h to 3 days (median, 95% CI, 12.0, 8–24 h). Forty-three (75.4%) cows had anorexia and 14 (24.6%) had a reduced appetite. Twenty-six (45.6%) had a history of colic before admission.

### General condition, abdominal contour and signs of pain

The general condition was mildly abnormal in 22.8% (13/57), moderately abnormal in 59.6% (34/57) and severely abnormal in 17.5% (10/57) of the cows. One cow was recumbent on admission. Ten cows (17.5%) had unilateral or bilateral abdominal dilatation, and 13 (22.8%) had nonspecific signs of pain including muscle tremors (19.3%, 11/57), piloerection (1.8%, 1/56) and bruxism (3.5%, 2/57). Twenty-six cows (46.4%, 26/56) had a tense abdominal wall. Twenty-five cows (43.9%) were in the colic phase but the clinical signs had not been recorded in detail in two cows. Colic manifested as treading (21.4%, 12/56), lordosis (21.4%, 12/56), restlessness (10.5%, 6/57), kicking (3.5%, 2/56) and sweating (3.6%, 2/56). Fifteen cows (26.3%) had one sign of colic, five (8.8%) had two, and three (5.3%) had three signs of colic, which were assessed as mild (38.6%, 22/57) or moderate (5.3%, 3/57).

### Heart and respiratory rates and rectal temperature (vital parameters)

Tachypnoea occurred in 50.9% (29/57), tachycardia in 42.1% (24/57) and decreased rectal temperature in 40.3% (23/57) of the cows (Table 1).

**Table 1:** Clinical findings in cows with paralytic ileus (means ± sd, medians, 95% CI, frequency distributions)

Variable	Finding		Number of cattle	%
Heart rate	Normal (60–80 bpm)		30	52.6
(*n* = 57, 76 bpm,	Decreased (52–59 bpm)		3	5.3
95% CI = 72–84 bpm)	Mildly increased (81–100 bpm)		16	28.1
	Moderately increased (101–120 bpm)		6	10.5
	Severely increased (128 bpm)		2	3.5
Rectal temperature	Normal (38.5–39.0 °C)		25	43.9
(*n* = 57, 38.6 °C,	Decreased (37.6–38.4 °C)		23	40.3
95% CI = 38.4–38.8 °C)	Increased (39.1–40.2 °C)		9	15.8
Respiratory rate	Normal (15–25 breaths per min.)		28	49.1
(*n* = 57, median = 28 breaths per min., 95% CI = 24–28 breaths per min.)	Increased (26–76 breaths per min.)		29	50.9
Rumen motility (*n* = 57)	Normal (2 or 3 strong contractions per 2 min.) Decreased Absent		10 29 18	17.5 50.9 31.6
Foreign body tests (*n* = 55)	All negative At least one test positive^1^ Equivocal		32 17 6	58.2 30.9 10.9
BSA and PSA on the left side (*n* = 57)	Both tests negative (normal) BSA positive		56 1	98.2 1.8
BSA and PSA on the right side (*n* = 57)	Both tests negative (normal) Only BSA positive Only PSA positive Both tests positive		10 20 5 22	17.5 35.1 8.8 38.6
Intestinal motility (*n* = 56)	Normal Decreased Absent		4 36 16	7.1 64.3 28.6
Transrectal findings^2^ (*n* = 56)	Normal findings Rumen dilated Dilated loops of small intestines Dilated small and large intestines Dilated intestines (small or large) Taut tissue bands Equivocal		12 16 24 3 5 7 6	21.4 28.6 42.9 5.4 8.9 12.5 10.7
Faeces, amount (*n* = 57)	Normal Faecal output reduced Rectum empty		3 43 11	5.3 75.4 19.3
Faeces, degree of comminution (*n* = 56)	Normal (well digested) Moderately digested Poorly digested Rectum empty		30 14 1 11	53.6 25.0 1.8 19.6
Faeces, consistency (*n* = 56)	Normal (pulpy) Thick pulpy Liquid Pasty Thin pulpy Rectum empty		23 13 4 3 2 11	41.1 23.2 7.1 5.4 3.6 19.3
Faeces, colour and abnormal contents in the rectum^3^ (*n* = 57)	Normal (olive) Dark Mucus Blood Fibrin Rectum empty		40 6 13 10 2 11	70.2 10.5 22.8 17.5 3.5 19.3

^1^ Positive: at least 3 of 4 attempts elicited a grunt

^2^ The total number of findings was 73 (130.4%) because 17 cows had more than one abnormal transrectal rectal finding

^3^ The total number of findings was 82 (143.8%) because 25 cows had more than one abnormal finding

BSA Ballottement and simultaneous auscultation

PSA Percussion and simultaneous auscultation

### Digestive tract abnormalities

**Fig. 1:**
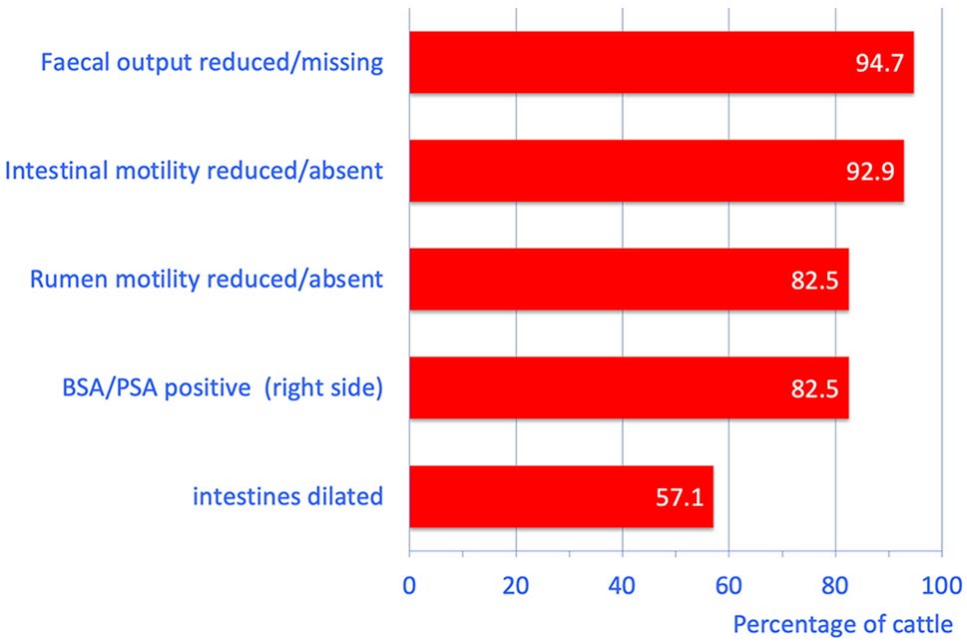
The most common digestive tract abnormalities found on clinical examination in 57 cows with paralytic ileus. BSA = Ballottement and simultaneous auscultation, PSA = Percussion and simultaneous auscultation

The most frequent abnormalities were little or no faecal output (94.7%, 54/57), reduced or absent intestinal motility (92.9%, 52/56), reduced or absent rumen motility (82.5%, 47/57), and positive ballottement and/or percussion and auscultation on the right side (82.5%, 47/57) (Fig. 1; Table 1). Transrectal palpation revealed dilated small and occasionally large intestines (57.1%, 32/56). Other abnormal findings included a positive result in at least one of the three foreign body tests in 30.9% (17/57) of the cows, and 12.5% (7/56) had taut mesenteric bands. The faeces were dark in 10.5% (6/57) of the cows and consistency varied from liquid to pulpy (normal) to thick pulpy and pasty. Abnormal faecal contents consisting of mucus, blood or fibrin were seen in 43.9% (25/57) of the cows.

### Other clinical findings

Other abnormal findings were reduced skin surface temperature (58.5%, 31/53), reduced skin elasticity (50.9%, 29/57), sunken eyes (43.9%, 25/57), dry and cool muzzle (43.9%, 25/57), moderately to severely hyperaemic scleral vessels (33.9%, 19/56), capillary refill time > 2 Sect. (30.4%, 17/56), pale mucous membranes (16.1%, 9/56), ammonia-like or otherwise foul breath (14.0%, 8/57) and droopy ears (5.3%, 3/57).

### Urinalysis

Urine pH ranged from 5.0 to 9.0 (median, 95% CI, 8.0, 8.0–8.0) and was acidic (5.0-6.9) in 23.2% (13/56) and alkaline (8.1-9.0) in 21.4% (12/56) of the cows. Specific gravity was decreased (< 1.020) in 22.7% (12/53) and increased (> 1.040) in 9.4% (5/53) of the cows. Glucosuria occurred in 56 (26.8%), haemoglobinuria/haematuria in 37.5% (21/56), ketonuria in 16.1% (9/56) and proteinuria in 1.8% (1/56) of the cows.

### Laboratory findings

**Fig. 2:**
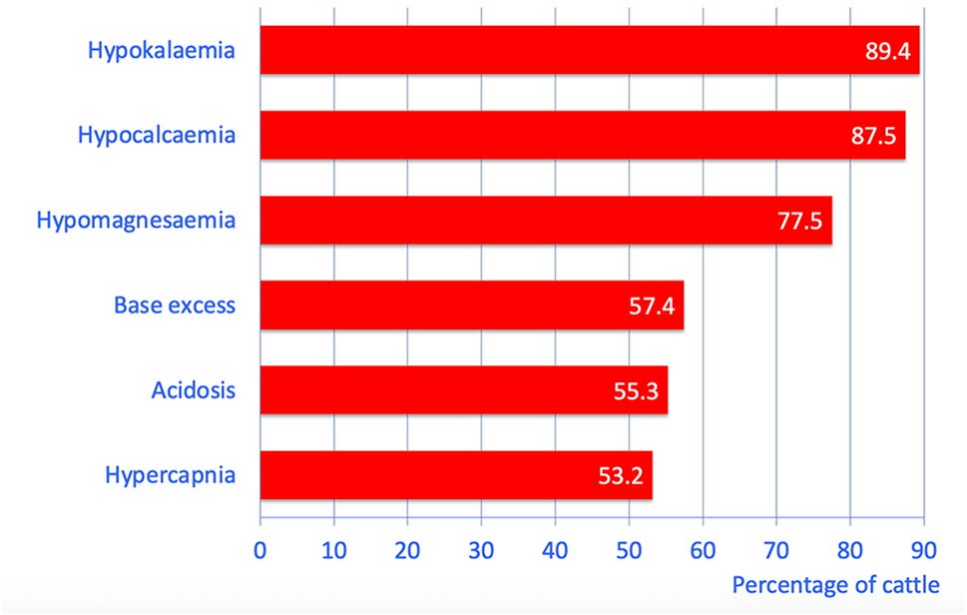
The most common abnormal blood variables in 57 cows with paralytic ileus. Acidosis was defined as a pH below 7.41

The principal abnormalities were hypokalaemia (89.4%, 51/57), hypocalcaemia (87.5%, 35/40), hypomagnesaemia (77.5%, 31/40), positive base excess (57.4%, 27/47), acidosis (55.3%, 26/47) and hypercapnia (53.2%, 25/47) (Fig. 2; Table 2). Less common or rare changes included azotaemia (42.1%, 24/57), increased activity of aspartate aminotransferase (42.1%, 24/57), hypochloraemia (42.1%, 24/57), haemoconcentration (40.4%, 23/57), hyperbilirubinaemia (40.4%, 23/57), hypophosphataemia (37.5%, 15/40), hyperproteinaemia (36.4%, 20/55), leukocytosis (35.1%, 20/57), increased concentration of blood bicarbonate (23.4%, 11/47), hyperfibrinogenaemia (20.4%, 11/54), increased activity of gamma-glutamyl transferase (15.8%, 9/57) and increased rumen chloride concentration (13.3%, 6/45).

**Table 2:** Laboratory findings in cows with paralytic ileus (means, medians, standard deviations, 95% CI, frequency distributions)

Variable (Mean ± sd, Median, 95% CI)	Finding	Number of cows	Percent
Haematocrit (*n* = 57), 34%, 95% CI = 33–37%	Normal (30–35%) Decreased (22–29%) Increased (36–52%)	28 6 23	49.1 10.5 40.4
Leukocytes (*n* = 57), 9,100 ± 3,729/µL, 95% CI = 8.110-10,090/µL	Normal (5,000–10,000/µL) Decreased (1,900-4,999/µL) Increased (10,001–18,300/µL)	29 8 20	50.9 14.0 35.1
Total protein (*n* = 55), 78.1 ± 11.0 g/L, 95% CI = 75–81 g/L	Normal (60–80 g/L) Decreased (48–59 g/L) Increased (81–108 g/L)	34 1 20	61.8 1.8 36.4
Fibrinogen (*n* = 54), 6.0 g/L, 95% CI = 5.0–6.0 g/L	Normal (4–7 g/L) Decreased (2.0–3.9 g/L) Increased (7.1–10.0 g/L)	36 7 11	66.7 12.9 20.4
Urea (*n* = 57), 6.1 mmol/L, 95% CI = 5.4–7.3 mmol/L	Normal (2.7–6.5 mmol/L) Increased (6.6–16.5 mmol/L)	33 24	57.9 42.1
Bilirubin (*n* = 57), 5.1 µmol/L, 95% CI = 3.8–7.2 µmol/L	Normal (0.8–6.5 µmol/L) Increased (6.6–19.9 µmol/L)	34 23	59.6 40.4
Calcium (*n* = 40), 1.7 mmol/L, 95% CI = 1.4–2.1 mmol/L	Normal (2.30–2.60 mmol/L) Decreased (1.00-2.29 mmol/L) Increased (2.61–3.15 mmol/L)	3 35 2	7.5 87.5 5.0
Magnesium (*n* = 40), 1.20 ± 0.29 mmol/L, 95% CI = 1.11–1.29mmol/L	Normal (0.80-1.00 mmol/L) Decreased (0.69–0.79 mmol/L) Increased (1.01–1.99 mmol/L)	6 3 31	15.0 7.5 77.5
Inorg. phosphate (*n* = 40), 1.50 ± 0.62 mmol/L, 95% CI = 1.30–1.69 mmol/L	Normal (1.30–2.40 mmol/L) Decreased (0.48–1.29 mmol/L) Increased (2.41–3.03 mmol/L)	21 15 4	52.5 37.5 10.0
Chloride (*n* = 57), 96 ± 7.7 mmol/L, 95% CI = 94–98mmol/L	Normal (96–105 mmol/L) Decreased (80–95 mmol/L) Increased (106–112 mmol/L)	30 24 3	52.6 42.1 5.3
Potassium (*n* = 57), 3.30 mmol/L, 95% CI = 3.10–3.50 mmol/L	Normal (4.0–5.0 mmol/L) Decreased (2.1–3.9 mmol/L) Increased (6.6 mmol/L)	5 51 1	8.8 89.4 1.8
AST (*n* = 57), 96 U/L, 95% CI = 90–111 U/L	Normal (25–103 U/L) Increased (104–874 U/L)	33 24	57.9 42.1
γ-GT (*n* = 57), 22 U/L, 95% CI = 20–24U/L	Normal (8–30 U/L) Increased (31–128 U/L)	48 9	84.2 15.8
Blood pH (*n* = 47), 7.41 ± 0.05, 95% CI = 7.39–7.42	Normal (7.41–7.45) Decreased (7.31–7.40) Increased (7.46–7.50)	11 26 10	23.4 55.3 22.3
pCO_2_ (*n* = 47), 45.2 ± 7.0 mmHg, 95% CI = 43-47mmHg	Normal (35.0–45.0 mmHg) Decreased (23.1–34.9 mmHg) Increased (45.1–63.4 mmHg)	18 4 25	38.3 8.5 53.2
Bicarbonate (*n* = 47), 27.3 ± 5.8 mmol/L, 95% CI = 26–29 mmol/L	Normal (20.0–30.0 mmol/L) Decreased (11.6–19.9 mmol/L) Increased (30.1–43.6 mmol/L)	34 2 11	72.3 4.3 23.4
Base excess (*n* = 47), 3.4 ± 6.0 mmol/L	Normal (-2 - +2 mmol/L) Decreased (-12.9 - -2.1mmol/L) Increased (+ 2.1 - +20.1 mmol/L)	14 6 27	29.8 12.8 57.4
Rumen chloride (*n* = 45), 20.0 mmol/L, 95% CI = 18–24 mmol/L	Normal (≤ 30 mmol/L) Increased (31–38 mmol/L)	39 6	86.7 13.3

AST Aspartate aminotransferase, γ-GT γ-glutamyltransferase (γ-GT)

### Ultrasonographic findings

The principal findings in the 50 cows that underwent ultrasonography were dilated small intestines with reduced or absent motility (Table 3). In 12 (24.0%) cows, parts of the large intestine (spiral colon, caecum) were also dilated and gas-filled. Fluid between the intestines was seen in 16.0% (8/50) of the cows, and 11 (22.0%) other cows had abomasal dilatation because of retrograde accumulation of ingesta.

**Table 3:** Ultrasonographic findings in 50 cows with paralytic ileus

Variable	Finding		Number of cows	%
Small intestines	Not dilated Dilated		0 50	0 100.0
Intestinal motility	Decreased Absent		24 26	48.0 52.0
Large intestine	Not dilated Dilated		38 12	76.0 24.0
Free fluid in the abdomen	No fluid visible Anechoic fluid without fibrin		42 8	84.0 16.0
Abomasum	Not dilated Dilated		39 11	78.0 22.0

### Diagnoses

Based on the initial clinical examination, a diagnosis could not be made in 21.0% (12/57) of the cows. One cow (1.8%) had a tentative diagnosis of right displaced abomasum, and in the remaining 44 (77.2%) cows a clinical tentative diagnosis of ileus was made. In 11 of the latter, PI was suspected because they also had acute mastitis (*n* = 1) or hypocalcaemia (*n* = 8). (Table 4). One of these 11 cows developed a PI after surgical correction of uterine torsion at a gestational age of 7 months, and another cow had a tentative diagnosis of PI because she had undergone successful conservative treatment for impaction of the spiral colon a year before.

**Table 4:** Findings and treatment in 11 non-operated cows with paralytic ileus

Case no.	Signalment	Main findings (in addition to signs of ileus)	Treatment
14	6.0-yr-old Brown Swiss cow	Gestational age 5 months, abomasal reflux syndrome, calcium = 1.37 mmol/L, potassium = 2.60 mmol/L	Glucose sodium chloride iv, calcium borogluconate iv, potassium chloride po, metoclopramide im
27	7.0-yr-old Brown Swiss cow	Gestational age 4 months, calcium = 1.13 mmol/L	Glucose and sodium chloride iv, calcium borogluconate iv, potassium chloride iv, potassium chloride po
29	3.7-yr-old Swiss Fleckvieh cow	Gestational age 7 months, uterine torsion, PI after surgical reduction, inorg. phosphate = 0.92 mmol/l	Glucose and sodium chloride iv, calcium borogluconate iv
30	3.5-yr-old Angus cow	Twin birth 2 days and acute mastitis one day before admission, recumbent. Pretreated with antibiotics and infusion of calcium	Glucose and sodium chloride iv, neostigmine iv, danofloxacin, iv flunixin meglumine iv
37	8.0-yr-old Brown Swiss cow	Fresh 4 months, calcium = 1.85 mmol/L, inorg. phosphate = 0.48 mmol/L	Glucose and sodium chloride iv, calcium borogluconate iv, sodium phosphate po, neostigmine iv
49	4.2-yr-old Brown Swiss cow	Gestational age 7 months, calcium = 1.96 mmol/L), inorg. phosphate = 0.85 mmol/L	Glucose and sodium chloride iv, calcium borogluconate iv; calcium, potassium chloride and sodium phosphate via orogastric tube
50	4.7-yr-old Brown Swiss cow	Fresh 70 days, sudden milk drop from 50 to 0 L, calcium = 1.37 mmol/L, potassium = 2.60 mmol/L)	Glucose and sodium chloride iv, calcium borogluconate iv; calcium and potassium chloride via orogastric tube
54	7.7-yr-old Swiss Fleckvieh cow	Fresh 2 weeks, calcium = 1.10 mmol/L, potassium = 2.40 mmol/L, fascioliasis, trichostrongyles and strongyles	Glucose and sodium chloride iv, calcium borogluconate iv; calcium and potassium chloride via orogastric tube
55	8.4-yr-old Brown Swiss cow	Gestational age 6 months, calcium = 1.19 mmol/L, inorg. phosphate = 0.90 mmol/L, potassium = 2.80 mmol/L	Glucose and sodium chloride iv, calcium borogluconate iv; calcium and potassium chloride via orogastric tube
56	7.3-yr-old Holstein cow	Fresh 6 days, one year ago treated because of obstipation of the spiral colon, calcium = 1.10 mmol/	Glucose and sodium chloride iv, calcium borogluconate iv; calcium and potassium chloride via orogastric tube
57	3.3-yr-old Swiss Fleckvieh cow	Fresh 3 weeks, calcium = 1.46 mmol/L, inorg. phosphate = 0.58 mmol/L	Glucose and sodium chloride iv, calcium borogluconate iv; calcium, potassium chloride and sodium phosphate via orogastric tube

### Concomitant diseases

Forty-four (77.2%) cows had one to three concomitant diseases including hypocalcaemia (42.1%, 24/57), gastrointestinal nematodes (31.6%, 18/57) and fascioliasis (8.8%, 5/57). Two each (3.5%) had a non-perforated intestinal ulcer, uterine torsion, enteritis and dictyocaulosis, and one each (1.8%) had fatty liver syndrome, retained placenta, a perforated intestinal ulcer, rumen acidosis, pyloric stenosis, abomasal paresis and acute mastitis. Two cows (no. 6 and 8) had a tentative diagnosis of abomasal ulcer type 1 because palpation of the abomasum during laparotomy elicited pain. Thirteen cows (22.8%) had no noticeable concomitant diseases.

### Overview of treatment

**Fig. 3:**
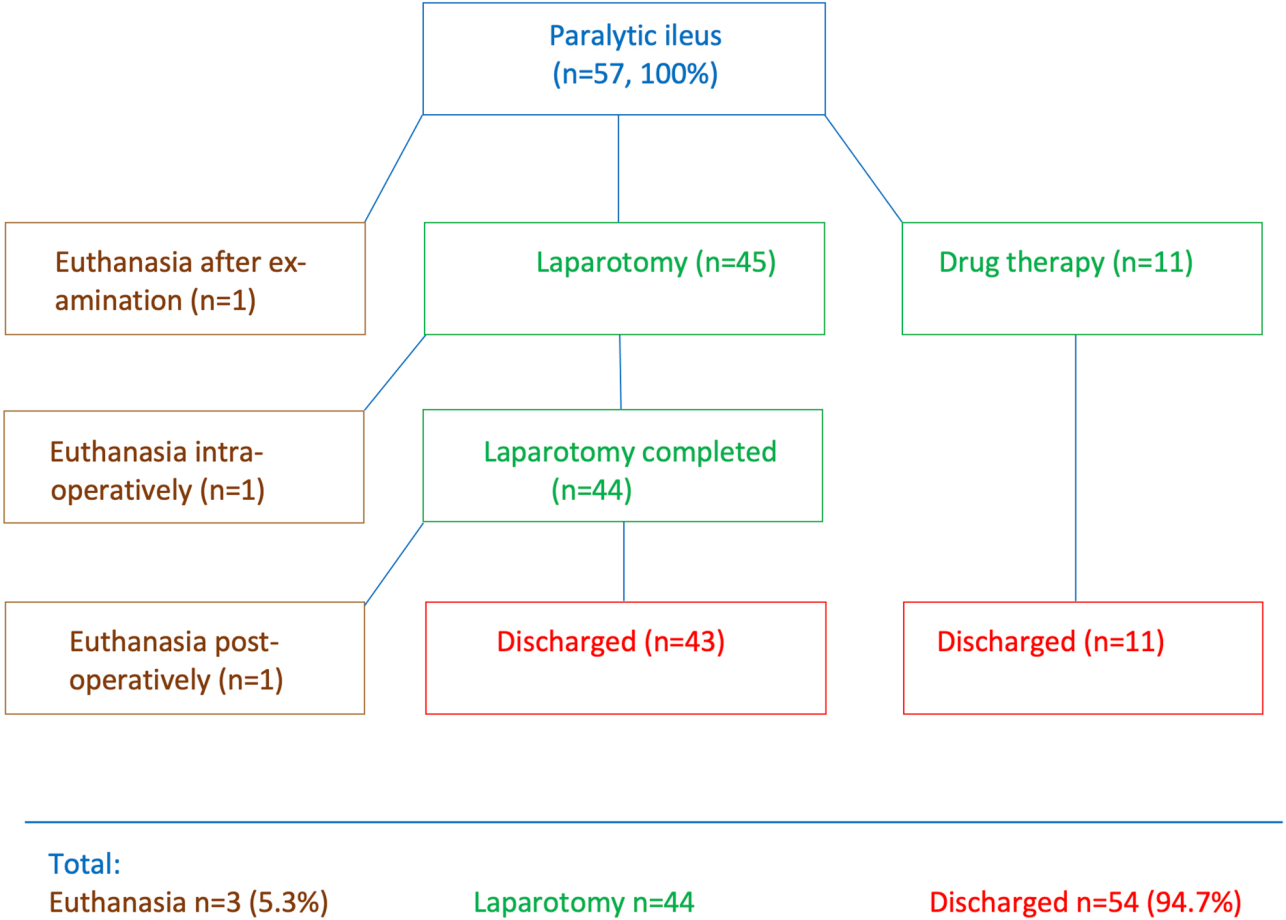
Treatment flowchart for 57 cattle with paralytic ileus

One cow was euthanized after the initial examination because the owner did not consent to the required surgery (Fig. 3). The 11 (19.3%) cows with a tentative diagnosis of PI were treated medically, and the remaining 45 (78.9%) underwent right flank laparotomy because mechanical ileus could not be ruled out. This was followed by medical treatment. Cow no. 4 was euthanized during the operation because of peritonitis caused by a perforated ulcer of the small intestine. Surgery was completed in 44 cows. One of the 44 (no. 18) was euthanized 4 days later, and postmortem examination showed more than 25 abomasal ulcers and erosions. Another cow (no. 21) at a gestational age of about 9 months was diagnosed with a 180-degree uterine torsion during laparotomy, which was corrected. The cow relapsed and required a second operation 2 days later, at which time the calf was delivered by caesarean section. In total, 43 of the operated cows and all 11 medically treated cows (54/57, 94.6%) were discharged, and 3 were euthanized after the initial examination (1), during laparotomy (1) or after the operation (1).

### Medical treatment of 11 cows with a tentative diagnosis of PI

Medical treatment included fluid therapy (11/11), antibiotics (2/11), pain medication (2/11), prokinetics (4/11), infusion of calcium borogluconate (11/11), oral calcium (6/11) and oral potassium chloride (8/11) (Table 4). All cows received 10 L of a solution containing 50 g glucose and 9 g sodium chloride per litre daily for 2 to 4 days administered as a slow intravenous drip via an indwelling jugular vein catheter. One cow with mastitis (no. 30) was treated with danoflocaxin (1.2 mg/kg) given intravenously, and another cow with uterine torsion received penicillin G procaine (12,000 IU/kg) given intramuscularly. The antibiotics were administered for 3 to 4 days. Two cows received flunixin meglumine (1 mg/kg) daily for 1 and 3 days. Prokinetic drugs were used in 3/11 cows for a duration of 2 (*n* = 3) or 5 days (*n* = 1). Two cows received neostigmine (40–45 mg) administered via continuous drip infusion for approximately 24 h, and one was treated with metoclopramide (30 mg) administered intramuscularly every 8 h for 9 treatments. All cows with hypocalcaemia received 500 to 1000 mL of 40% calcium borogluconate intravenously (duration of the infusion ≈ 60 min) for 1 to 5 days (median, 95% CI, 2, 1–3 days) and 7 cows also received oral calcium (Calcivet, Provet, Lyssach) administered via an orogastric tube for 1 to 3 days until normocalcaemia (Ca ≥ 2.30 mmol/L) occurred. Eight cows with hypokalaemia were treated (also via an orogastric tube) with daily oral doses of 60 to 100 g of potassium chloride for 1 to 5 days (median, 95% CI, 2.5, 1–5 days) and 4 cows with hypophosphataemia were treated with 350 g of sodium phosphate for 1 to 2 days until normokalaemia (K ≥ 4 mmol/L) and normophosphataemia (anorg. *P* ≥ 1.30 mmol/L) occurred.

### Surgical findings and postoperative treatment

Forty-four cows underwent a standing laparotomy and one was operated in lateral recumbency. In all cows, PI involved the small intestine, and in four, the large intestine as well (this includes the 45 operated cows and the cow that was euthanized after the initial examination but not the 11 cows that only received medical treatment). In 38 cows, the only findings were intestinal dilatation and slight bluish discolouration of the intestine. Six cows had mild to moderate serositis, and one cow had severe inflammatory changes because of a ruptured intestinal ulcer. Five cows had mesenteric oedema, five had adhesions and five had an increased amount of peritoneal fluid. In addition, a markedly hard omasum (1), a painful and distended abomasum (1), uterine torsion (1), and a pedunculated mass originating from the peritoneum of the ventral abdominal wall (1) were found; the latter was not thoroughly examined.

Nineteen of the 44 cows underwent exploratory laparotomy with no additional therapeutic measures. In 18 cows, the small intestine was massaged, six underwent caecotomy and small intestinal massage, and reduction of a uterine torsion was done in one other. One cow at a gestational age of about 9 months was diagnosed with a 180-degree uterine torsion during laparotomy, which was corrected (see also Overview of treatment). The cow relapsed and required a second operation 2 days later, at which time the calf was delivered by caesarean section.

Postoperative medical treatment of the 43 cows that were operated successfully and subsequently discharged included fluid therapy (43/43), antibiotics (43/43), pain control (42/43), prokinetic drugs (38/43), potassium chloride (22/43) and calcium borogluconate (23/43). The cows received 10 L of a solution containing 50 g glucose and 9 g sodium chloride per litre daily for 2 to 5 days (median, 3 days) (details of doses and administration see treatment of the 11 non-operated cows). Antibiotic treatment included penicillin G procaine (12,000 IU/kg) given intramuscularly (38/43), danofloxacin (1.2 mg/kg) given intravenously (3/43), oxytetracycline (5 mg/kg) given intravenously (1/43) and amoxicillin (7 mg/kg) given intramuscularly (1/43). The antibiotic treatment was administered for 2 to 6 days (median, 95% CI, 4, 4 days). All but one cow received daily flunixin meglumine (1 mg/kg), ketoprofen (3 mg/kg), metamizole (35 mg/kg) or a combination of flunixin meglumine and metamizole given intravenously for 1 to 5 days (median, 95% CI, 3, 3 days). Prokinetic drugs were used in 38/43 cows for 1 to 5 days (median, 95% CI, 3, 3 days). Eighteen cows received neostigmine (40–45 mg) administered via continuous drip infusion, and 19 received intramuscular metoclopramide (30 mg), typically seven to nine times at eight-hour intervals, and one cow received both drugs. Twenty-three cows with hypocalcaemia were treated once a day with 500 mL of 40% calcium borogluconate intravenously for a duration of 1 to 4 days (median, 95% CI, 1, 1–3 days), and 22 cows with hypokalaemia were treated with daily oral doses of 60 to 100 g of potassium chloride for 1 to 3 days (median, 95% CI, 1, 1–2 days) until normokalaemia occurred.

### Postmortem findings

All three euthanized cows had dilated intestines in the absence of a mechanical cause. In addition, two cows had multiple abomasal ulcers, one had peritonitis and one had a perforated jejunal ulcer.

### Further disease progression

The general condition of 53 of the 54 discharged cows normalised within 1 to 6 days (median, 95% CI, 2, 1–2 days), the appetite of 53 cows within 1 to 5 days (2, 2–3 days), and defaecation of all cows within 1 day (1, 1–1 day) of the operation. The rectal temperature of the 54 surviving cows did not change significantly between admission (median, 95% CI, 38.6 °C, 38.4–38.7 °C) and day 7 (38.6, 38.4–38.7 °C). The same was true for the heart rate, which ranged from 76 (95% CI, 72–84 bpm) at admission to 62 bpm (60–84 bpm) at day 7. Fifty-four of the 57 cows (94.7%) were discharged 3 to 7 days and one cow 10 days (median, 5 days) after admission.

### Long-term outcome for the 54 successfully treated cows

The long-term outcome was determined 2 years after discharge via telephone interview. Of the 54 discharged cows, milk production and reproductive performance were good in 25. Three cows had been slaughtered because of recurrence of PI and eight others because of economic or other health reasons. The outcome was not known for the remaining 18 cows.

## Discussion

The abnormal clinical findings in cows with PI were analogous to those of other forms of ileus but they differed in frequency. Twenty-five (43.9%) cows with PI had colic, which was presumably caused by dilatation of the small intestine. Cows with small intestinal strangulation (40.0%, 24/60) [15], and small intestinal intussusception (46.8%, 59/126) [9] had similar frequencies of colic, in contrast to cows with small intestinal volvulus (66.0%, 31/47) [16], mesenteric torsion (65.6%, 40/61) [11] and ileal impaction (68.2% 15/22) [17], which had considerably higher frequencies. The mesentery is particularly sensitive to pain but is less likely to be affected in cows with PI compared with other forms of ileus. Other important clinical findings including tachycardia, intestinal atony, positive ballottement and/or percussion and auscultation on the right side, no faecal output and dilated small intestine palpated transrectally differed mostly in frequency of occurrence but not in their manifestation. Therefore, it was not possible to definitively diagnose PI based on the results of clinical examination. Likewise, ultrasonography did not differentiate PI from other forms of ileus because dilated small intestine and impaired intestinal motility are also typical for mechanical ileus [12].

In cows with clinical signs of ileus, PI can only be diagnosed when a mechanical ileus is ruled out, but this is almost impossible without laparotomy. Paralytic ileus should be part of a differential diagnosis in cows with concurrent signs of acute illness, which occurred in 11 cows in the present study. Nine of the 11 cows had severe hypocalcaemia, which was considered a cause of PI by other authors [2, 3], one had acute mastitis and one other had uterine torsion. Another cow had a tentative diagnosis of PI because she had undergone successful conservative treatment for impaction of the spiral colon a year before.

The biggest challenge in the treatment of PI is deciding whether medical treatment or laparotomy is best. Unfortunately, sound decision-making guidelines are not available for this. In our view, veterinarians must be convinced that the ileus is functional to institute medical treatment alone. For example, the authors feel that cows with severe hypocalcaemia and no indication of mechanical ileus can initially be treated medically. The same applies to cows with other primary diseases including acute mastitis or uterine torsion. However, an indispensable prerequisite for this approach is intensive monitoring of the cow and preparedness for possible laparotomy; prompt surgical treatment in cases that deteriorate or do not respond to medical treatment is imperative. The consequences are usually disastrous when cows with mechanical ileus are treated conservatively for 12 h or longer because of the risk of intestinal rupture and generalised peritonitis. Defaecation after calcium infusion and/or an increase in appetite are positive signs. Conservative treatment consists primarily of pain medication, calcium and fluid therapy; in practice, cows should receive pain medication and calcium first. Prokinetics are contraindicated in cases in which mechanical ileus cannot be ruled out because they can lead to intestinal rupture.

The main purpose of laparotomy in a cow with a tentative diagnosis of PI is to confirm the diagnosis; it is not required for treatment, in contrast to mechanical ileus. In our opinion, it is better to err on the side of caution when in doubt because a timely laparotomy carried out *lege artis* does not usually affect the patient negatively [13]. When a cow cannot be closely monitored after initiation of conservative treatment, laparotomy is indicated. Other indications for surgery are no response to treatment, deterioration in general condition, ongoing colic and no faecal output after treatment. Cows that have been treated unsuccessfully with calcium before admission, severely ill cows, those with a heart rate over 100 bpm or with severe colic, and those with pronounced positive ballottement and/or percussion and auscultation should receive prompt surgical treatment. Mucus and/or blood in the rectum does not necessarily indicate a mechanical ileus because these were also seen in 22.8 and 17.5% of cows with PI. Mucus or blood in the rectum occurred at similar frequency in cows with incarceration (15.3/12.9%), volvulus (19.1/14.9%), and strangulation (21.6/16.7%), but was more common in cows with intussusception (32.5/46.0%).

Whether the 11 conservatively treated cows had PI cannot be answered conclusively. Laparotomy and/or postmortem examination have been used as the gold standard for diagnosis in our previous studies on ileus. However, neither of these diagnostic tools was necessary because all 11 cows responded to treatment. Based on our strict diagnostic criteria and our extensive experience with bovine gastrointestinal diseases, we feel that our tentative diagnoses were correct. With mechanical ileus, the cows would not have responded to conservative treatment and their health would have continued to deteriorate. We debated whether to publish the findings of these cows but ultimately concluded that they would add to the study.

Based on a discharge rate of 94.7%, the prognosis in cows with PI appears to be very good and better than that of other types of ileus. The discharge rate was 23.0% (14/61) for cows with mesenteric torsion [11], 38.3% (18/47) for small intestinal volvulus [16], 38.8% (33/85) for incarceration [18], 44.4% (56/126) for intussusception [9] and 55.6% (10/18) for internal herniation [19]. Discharge rates similar to PI occurred in cows with strangulation (81.7%, 49/60) [15] and an ever higher rate was recorded for cows with ileal impaction (100%, 22/22) [17]. The different discharge rates reflect the different severities of intestinal changes with different types of ileus.

## Conclusions

Compared with other forms of ileus, the prognosis of PI is favourable provided that underlying diseases can be treated successfully. The most challenging factor is the differentiation of mechanical and functional ileus using non-invasive procedures. Medical treatment alone should only be used when cows can be intensively monitored and prompt surgical treatment can be carried out if necessary. Conservative treatment should include intravenous fluids, calcium infusion and analgesics but not prokinetics. Laparotomy is indicated when the cow’s condition does not improve within a few hours.

## Data Availability

The datasets used and analysed for this study are available from the corresponding author on reasonable request.
